# Application of reverse-phase HPLC to quantify oligopeptide acetylation eliminates interference from unspecific acetyl CoA hydrolysis

**DOI:** 10.1186/1753-6561-3-S6-S5

**Published:** 2009-08-04

**Authors:** Rune Evjenth, Kristine Hole, Mathias Ziegler, Johan R Lillehaug

**Affiliations:** 1 Department of Molecular Biology, University of Bergen, N-5020 Bergen, Norway; 2 Department of Surgical Sciences, University of Bergen, N-5020 Bergen, Norway; 3 Department of Surgery, Haukeland University Hospital, N-5021 Bergen, Norway

## Abstract

Protein acetylation is a common modification that plays a central role in several cellular processes. The most widely used methods to study these modifications are either based on the detection of radioactively acetylated oligopetide products or an enzyme-coupled reaction measuring conversion of the acetyl donor acetyl CoA to the product CoASH. Due to several disadvantages of these methods, we designed a new method to study oligopeptide acetylation. Based on reverse phase HPLC we detect both reaction products in a highly robust and reproducible way. The method reported here is also fully compatible with subsequent product analysis, e.g. by mass spectroscopy. The catalytic subunit, hNaa30p, of the human NatC protein N-acetyltransferase complex was used for N-terminal oligopeptide acetylation. We show that unacetylated and acetylated oligopeptides can be efficiently separated and quantified by the HPLC-based analysis. The method is highly reproducible and enables reliable quantification of both substrates and products. It is therefore well-suited to determine kinetic parameters of acetyltransferases.

## Background

Acetylation of proteins is a common protein modification that occurs either in the N-terminal α amino group (N^α^-acetylation) or the ε amino group of lysine residues (N^ε^-acetylation). The corresponding acetylation reactions are catalysed by N^α^-acetyltransferases (NATs) or histone acetyltransferases/lysine acetyltransferases (HATs/KATs), respectively [1,2].

The important biological functions of protein acetylation have promoted extensive functional studies of different acetyltransferases to determine their kinetic properties, substrate specificities and catalytic mechanisms. For most of the enzymatic analyses, two different kinds of acetyl transfer assays are used. One uses radioactively labelled acetyl CoA as substrate [3]. The generation of radioactively labelled oligopeptides is monitored by a filter-binding assay and liquid scintillation counting [3]. This assay is very sensitive [4], but due to the use of radioactivity, the assay represents potential environmental and health risks and it is therefore relatively demanding to perform due to safety precautions. The other commonly used method is a spectrophotometric assay that continuously measures the amount of CoASH generated by the acetyltransferase reactions [5]. The CoASH is determined by a coupled enzyme system using either α-ketoglutarate dehydrogenase or pyruvate dehydrogenase. The CoASH dependent oxidation of α-ketoglutarate or pyruvate is coupled to the reduction of NAD^+ ^to NADH, which is determined spectrophotometrically at 340 nm. This assay is relatively inexpensive and can be performed with standard spectrophotometric equipment. A disadvantage of both methods is the difficulty to detect whether an oligopeptide substrate contains more than one lysine target residue.

Using the production of CoA as the basis for measuring acetyltransferase activity is linked to another potentially severe cause of error. In nearly all KAT assays, measuring the N^ε^-acetylation of lysines, a substantial amount of acetyl CoA will spontaneously react with the ε amino group on lysine side chains, making it necessary to design proper controls to correct for this effect [4].

In addition, using the CoA-NADH coupled enzyme assay, it is not possible to use CoA as inhibitor to study acetyltransferase catalytic mechanism and, similarly to the filter assay, when more than one lysine target is present in the oligopeptide substrate, detailed acetylation site specificity can not be studied. To determine Km and Vmax values, the initial reaction rate must be determined under conditions giving linear initial reaction rates over the range of substrate concentration used, normally the substrate conversion should not exceed 10 – 15% [4]. Furthermore, build-up of high concentrations of products may cause product inhibition. It is therefore of interest to obtain detailed information both on substrate consumption and product production. The radioactivity-based filter assay and the coupled-enzyme assay do not provide information on product consumption and detailed control experiments must be added. In the present article, we present a simple method for studying oligopeptide acetylation, using reverse phase HPLC detecting acetylated oligopeptides, in addition to CoASH. This method uses semi-automated HPLC-technology providing a fast, sensitive and highly reproducible assay for protein acetyltransferases. The instrument records the UV spectrum between 200 and 300 nm, thereby enabling to monitor acetyl CoA and CoA (260 nm) and the peptide substrate and acetylated oligopeptides (215 nm) simultaneously. In addition, a radioactive flow detector may be connected to detect ^14^C/^3^H-acetyl-oligopeptides to increase sensitivity, if required.

After the peptide separation using reverse phase HPLC, the relevant absorbance peaks are integrated and the corresponding areas are converted to amounts of product formed. The continuous recording of the UV spectra allows for each peak to be evaluated for the maximum absorbance wavelength which can be used to determine the purity of each absorbance signal.

Since the oligopeptide separation is performed in buffers containing acetonitrile and TFA, the HPLC method is also fully compatible with subsequent analytical procedures such as mass spectrometry to determine the site of modification.

## Methods

### Chemicals

Chemicals used in this study that are important for correct enzymatic determination are acetonitrile – ACN (Merck), trifluoracetic acid – TFA (Roche), acetyl CoA (Sigma), [1-^14^C] acetyl CoA (56 mCi/mmol – GE Healthcare). All reagents were of analytical grade. Custom made oligopeptides (Table 1) were purchased from BioGenes, Germany.

**Table 1:** Oligopeptides used as substrates in this study

**Oligopeptide sequence**	**Abbreviation**	**Protein name**^¤^
[H]**MLGTEGG **RWGRPVGRRRRPVRVYP [OH]*	_1_MLGTE-RRR_24_	hnRNP H; (P31943)
[H]**MLALISR **RWGRPVGRRRRPVRVYP [OH]*	_1_MLAL-RRR_24_	hARL8b; (Q9NVJ2)
[H]**MLGTGPA **RWGRPVGRRRRPVRVYP [OH]*	_1_MLGTG-RRR_24_	mTOR; (P42345)
[H]**MLGPEGG **RWGRPVGRRRRPVRVYP [OH]*	_1_MLGP-RRR_24_	hnRNP F; (P52597)

* Bold indicates amino acids found in corresponding protein while abbreviation indicate the names used in the text. The amino acid residues 8 to 24 of the oligopeptides are annotated 'RRR' in the text. ^¤^Name of protein with an N-terminal sequence identical to the seven first amino acids of the oligopeptide.

### Cloning, expression and purification of MBP-hNaa30p

The catalytic subunit of the human NatC complex; hNaa30p (earlier known as hMak3), a NAT acetyltransferase, was used to establish the reverse phase HPLC method. h*NAA30 *was cloned into the prokaryotic expression system pETM-MBP (originally obtained from G. Stier – EMBL, Heidelberg, Germany) and expressed in *E. coli*. The cloning, expression and purification were performed as described [6].

### Separation of non acetylated and acetylated oligopeptides using reverse phase HPLC

**Figure 1:**
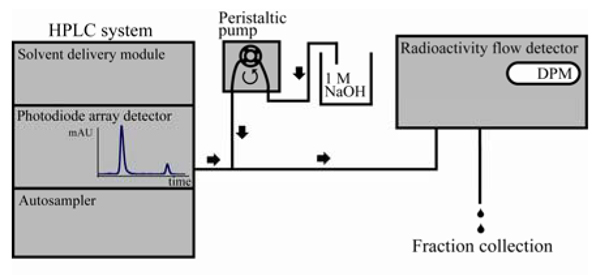
**Schematic presentation of the HPLC system included the equipment needed to detect the radioactivity**.

The acetylation activity was analysed by a reverse phase HPLC system, consisting of a LC-20AB solvent delivery module, an SPD-M20A photodiode array detector and a SIL-20AC autosampler (Shimadzu Prominence), and a 250 mm × 3 mm Nucleosil C18 HD column (Macherey-Nagel) reverse phase HPLC column. In addition, a radioactivity flow detector (LB 509 – Berthold) and a peristaltic pump were connected down stream of the HPLC absorbance detector (Figure 1). All absorbance signals were quantified by integrating the peak of interest using the software LCSolution Version 1.21 SP1.

Prior to sample injection, the column was equilibrated for 5 minutes (0.35 ml/min flow rate) with buffer A (5% acetonitrile (ACN) and 0.1% trifluoracetic acid (TFA)). After sample injection, the column was washed for 8 minutes with 2% elutionbuffer B (90% ACN, 0.1% TFA). The oligopeptides were then eluted employing a 40 minutes linear gradient from 2% to 40% buffer B. The column was then rinsed with 95% buffer B for 5 minutes. Finally, a 2 minutes linear gradient to 2% buffer B was performed.

### Determination of steady-state kinetic constants with reverse phase HPLC

80 nM of purified MBP-hNaa30p with 200 μM _1_MLGTE-RRR_24 _oligopeptide and 300 μM acetyl CoA in acetylation buffer (50 mM Tris-HCl (pH 8.5), 10% Glycerol, 1 mM EDTA) were incubated for 60 minutes at 37°C. Samples were collected after 0, 10, 20, 30, and 60 minutes incubation and analyzed by reverse phase HPLC.

To determine the K_m oligopeptides_, 80 nM of purified MBP-hNaa30p was incubated with varying concentrations of oligopeptides (30 to 350 μM) and 300 μM acetyl CoA in acetylation buffer for 30 minutes at 37°C. When determining the K_m acetyl CoA_, 300 μM of _1_MLAL-RRR_24 _peptide was used in combination with varying ‘concentrations of acetyl CoA (4 to 40 µM). The enzyme reactions were stopped by adding TFA to final concentrations of 1% (v/v). The amounts of acetylated oligopeptides were determined based on the absorbance at 215 nm, while the production of CoA was determined by using the absorbance at 260 nm. The steady-state enzyme kinetic parameters were calculated by nonlinear regression analysis using the SigmaPlot Technical Graphing Software (SPSS Inc.) The normality tests for all Km determinations were passed with value > 0.8.

To verify the elution time for the acetylated oligopeptides, we conducted a time dependent acetylation assay by incubating purified MBP-hNaa30p (80 nM) with the oligopeptide _1_MLGTE-RRR_24 _(200 μM) and [1-^14^C] acetyl CoA (final concentration 300 μM with specific activity 11.2 mCi/mmol). Samples were collected after 0, 10, 20, 30, and 60 minutes, placed on ice and adjusted to 1% TFA.

## Results

### The HPLC system

**Figure 2:**
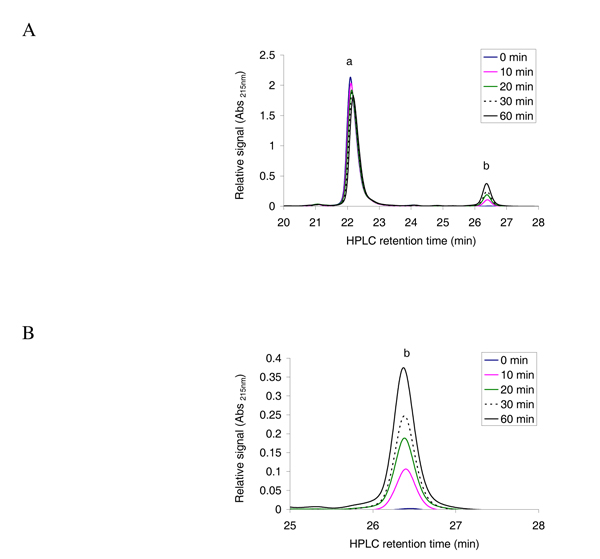
**Reverse phase HPLC absorbance profile at 215 nm for the separation of acetylated and non-acetylated peptides**. **A**; The oligopeptide _1_MLGTE-RRR_24 _(200 μM) was incubated with acetyl CoA (300 μM) and purified MBP-hNaa30p (80 nM) in acetylation buffer for 60 minutes at 37°C. Samples were collected at indicated time points and analysed with reverse phase HPLC. The resulting absorbance profile at 215 nm indicate good separation of unacetylated ('a') and acetylated oligopeptides ('b'). **B**; An expanded version of the absorbance profile for the formation of acetylated oligopeptide. A clear time dependent increase in the absorption signal is observed.

**Figure 3:**
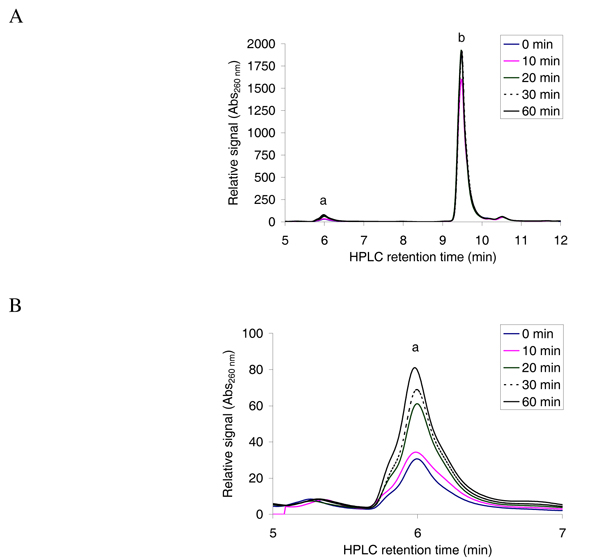
**Reverse phase HPLC absorbance profile of the separation of CoA and acetyl CoA**. **A**; The oligopeptide _1_MLGTE-RRR_24 _(200 μM) was incubated with acetyl CoA (300 μM) and purified MBP-hNaa30p (80 nM) in acetylation buffer for 60 minutes at 37°C. Samples were collected at indicated time points and analysed with reverse phase HPLC. The resulting absorbance profile at 260 nm indicates good separation of CoA ('a') and acetyl CoA ('b'). **B**; An expanded version of the absorbance profile for the formation of CoA. A time dependent increase in the absorption signal is observed.

To establish the chromatographic procedure, first, the elution times for unmodified oligopeptides were determined by injecting 3 nmol of pure oligopeptides diluted in the acetylation buffer on the HPLC system. Then we tested whether unacetylated and acetylated oligopeptides could be efficiently separated. We incubated an oligopeptide that is expected be a good hNaa30p substrate [7]; _1_MLGTE-RRR_24 _(200 μM) and 300 μM acetyl CoA with 80 nM of purified MBP-hNaa30p in the acetylation buffer. The sample was incubated at 37°C with aliquots collected after 0, 10, 20, 30, and 60 minutes (see Methods). From the elution profile at Abs 215 nm, we observed, as expected, a large amount of unmodified oligopeptide (Figure 2A, peak annotated 'a') and an additional peak with delayed elution time which increased with enzyme reaction time (Figure 2A and 2B, peak annotated 'b'). The changes in absorbance profiles recorded at 260 nm, detecting CoA and acetyl CoA in the same run, showed the same tendencies as the Abs 215 nm signals (Figure 3A and 3B). We observed a large excess of acetyl CoA (Figure 3A, peak annotated 'b') and an additional peak that increases during the course of the reaction (Figure 3A and 3B, peak annotated 'a'). The elution times of acetyl CoA and CoA were determined by injecting 3 nmol of CoA or acetyl CoA and recording the resulting absorbance profiles. This analysis showed that CoA and acetyl CoA eluted after 6 minutes and 9 minutes and 30 seconds, respectively, peaks 'a' and 'b' in Figure 3A.

### Analysing the sensitivity of the method

**Figure 4:**
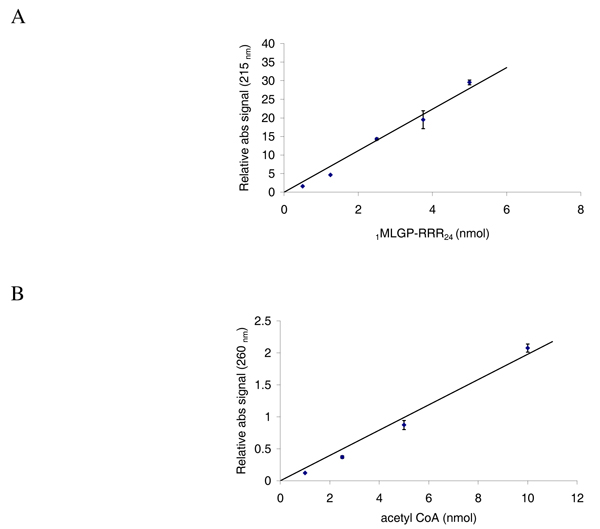
**Standard curves of increasing amount of the substrate _1_MLGP-RRR_24_ and acetyl CoA analysed by reverse phase HPLC**. **A**; Five different amounts of the oligopeptide 1MLGP-RRR24, diluted in acetylation buffer, were analysed by reverse phase HPLC. The resulting absorption signals at 215 nm were quantified. Each amount were analysed three times and error bars indicate S.D. **B**; Five different amounts of acetyl CoA, diluted in acetylation buffer, were analysed by reverse phase HPLC. The resulting absorption signals at 260 nm were quantified. Each amount were analysed three times and error bars indicate S.D.

The sensitivity of the HPLC based analysis was studied by injecting different amounts of oligopeptide and recording the resulting Abs 215 nm signal. The sensitivity for acetyl CoA was determined by injecting different amounts of acetyl CoA and recording the resulting Abs 260 nm signal. A linear correlation between the absorbance signals and the amount of substrates added was observed (Figure 4A and 4B). We noted that 0.5 nmol was the lower limit for reliable quantification of oligopeptides at 215 nm, which corresponded to 5 μM in a reaction volume of 100 μl.

**Figure 5:**
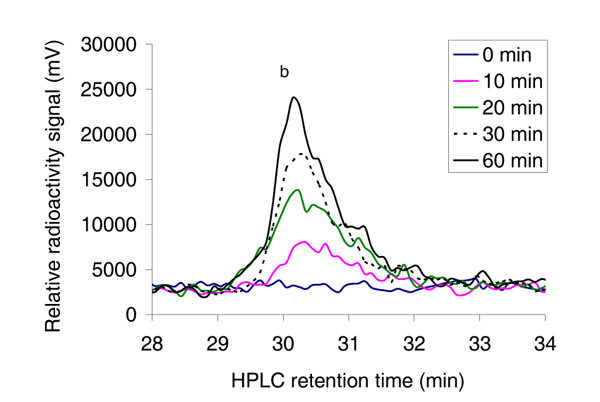
**The radioactive signal from oligopeptides being modified with radioactive acetyl CoA**. The oligopeptide _1_MLGTE-RRR_24 _(200 μM) was incubated with [1-^14^C] acetyl CoA (final 300 μM with specific activity 11.2 mCi/mmol) and purified MBP-hNaa30p (80 nM) in acetylation buffer for 60 minutes at 37°C. Samples were collected at indicated time points and analysed with reverse phase HPLC that had been connected to a radioactivity flow detector after the absorbance detector. A clear time dependent increase in the radioactivity signal is observed, verifying that the eluted oligopeptides are labelled with radioactive acetyl groups.

To enhance the detection sensitivity and to verify that the novel delayed absorption peak represented oligopeptides with one added acetyl group, we connected a radioactive flow detector after the absorbance detector (Figure 1). This allowed us to use radioactively labelled acetyl CoA as acetyl donor. To prevent background accumulation of radioactivity in the solid scintillator detector system, 1 M NaOH was mixed to the column effluent in a 1:10 mixing ratio by connecting a peristaltic pump in-line between the absorbance detector and the radioactivity flow detector (Figure 1). High concentration of NaOH was used so that the dilution of the samples, thus dilution of the radioactive signals was as low as possible. Using [1-^14^C] acetyl CoA as acetyl donor in the reaction, we observed a similar increase in radioactively labelled acetylated oligopeptides (Figure 5) as observed for the Abs 215 nm absorption signal (Figure 2B, peak 'b'). This verifies that the peptide with delayed elution time is labelled with radioactive acetyl groups and that the quantity of the signal increased with enzyme reaction time.

### Quantification of CoA has the potential of generating false kinetic data

**Figure 6:**
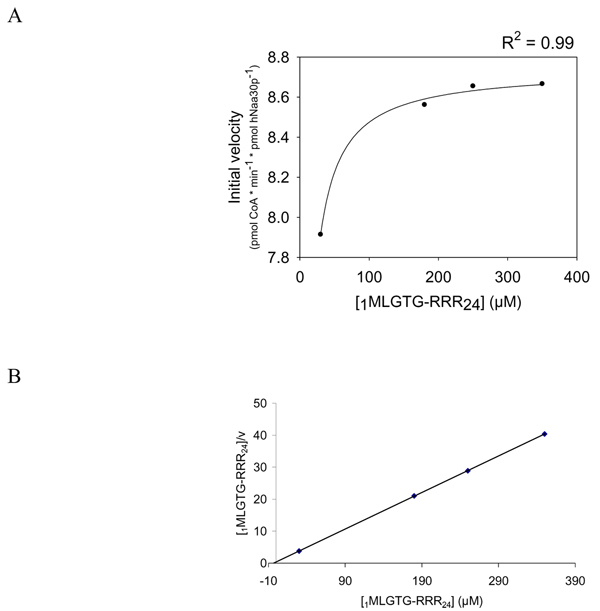
**Determination of Km and Vmax for the _1_MLGTG-RRR_24 _oligopeptide, based on the generation of CoA**. Purified MBP-hNaa30p (80 nM) was incubated with 300 μM acetyl CoA and varying concentrations of _1_MLGTG-RRR_24 _(30 – 350 μM) in acetylation buffer for 30 minutes at 37°C. **A**; Non linear regression analysis of the dose dependent curve generated based on the analysis of the CoA absorption signal at 260 nm. The coefficient of determination (R^2^) is given above the plot. **B**; Hanes-Woolf plot of the dose dependent acetylation signal.

**Figure 7:**
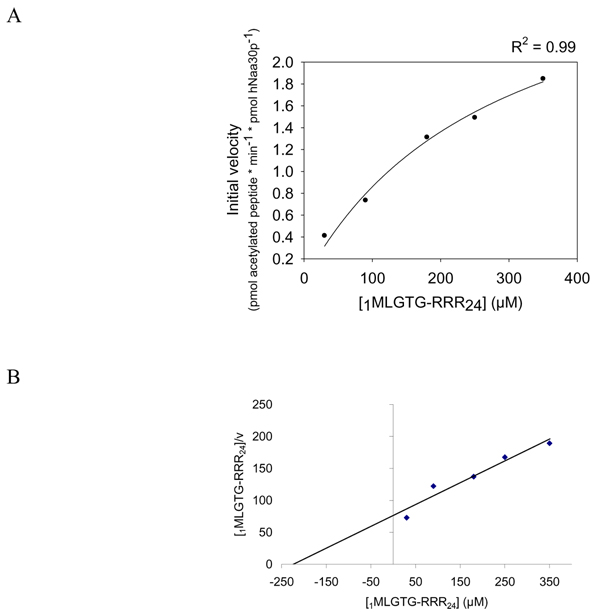
**Determination of Km and Vmax for the _1_MLGTG-RRR_24 _oligopeptide, based on the generation of acetylated _1_MLGTG-RRR_24 _oligopeptide**. Purified MBP-hNaa30p (80 nM) was incubated with 300 μM acetyl CoA and varying concentrations of _1_MLGTG-RRR_24 _(30 – 350 μM) in acetylation buffer for 30 minutes at 37°C. **A**; Non linear regression analysis of the dose dependent curve generated based on the analysis of the acetylated oligopeptide absorption signal at 215 nm. The coefficient of determination (R^2^) is given above the plot. **B**; Hanes-Woolf plot of the dose dependent acetylation signal.

**Figure 8:**
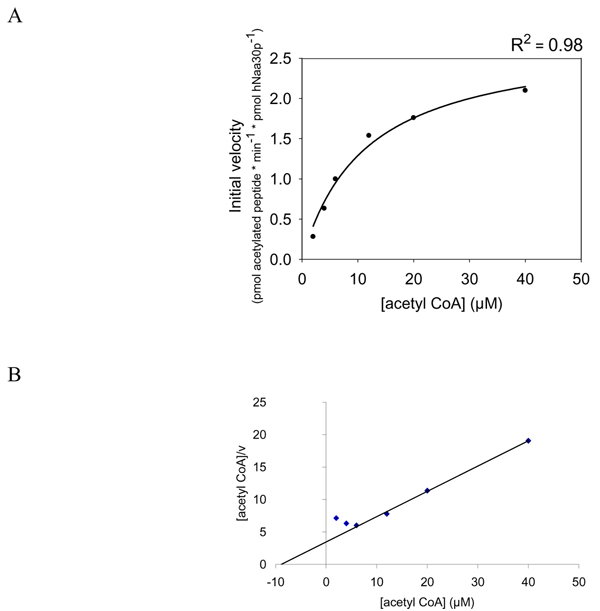
**Determination of Km and Vmax for acetyl CoA, based on the generation of acetylated _1_MLAL-RRR_24 _oligopeptide**. Purified MBP-hNaa30p (80 nM) was incubated with _1_MLAL-RRR_24 _oligopeptide at saturated levels and varying concentrations of acetyl CoA (4 – 40 μM) in acetylation buffer for 30 minutes at 37°C. **A**; Non linear regression analysis of the dose dependent curve generated based on the analysis of the acetylated oligopeptide absorption signal at 215 nm. The coefficient of determination (R^2^) is given above the plot. **B**; Hanes-Woolf plot of the dose dependent acetylation signal.

**Figure 9:**
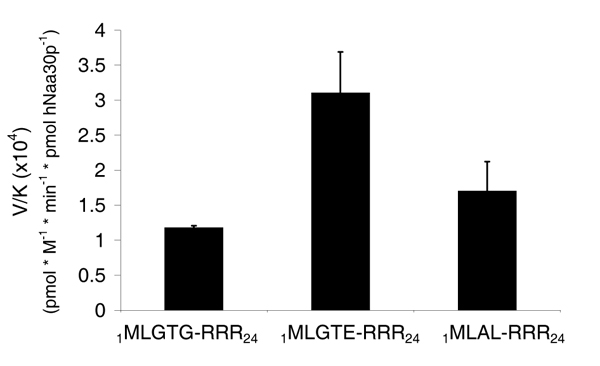
**MBP-hNaa30p specificity constants (V/K) for selected oligopeptides based on detection of acetylated oligopeptides_(215 nm)_**. Purified MBP-hNaa30p (80 nM) was incubated with selected oligopeptides and acetyl CoA (300 μM) in acetylation buffer for 30 minutes at 37°C. The acetylation kinetics was analysed by reverse phase HPLC. V/K is the V_max_/K_m (oligopeptides)_. Error bars indicate S.D. Experiments are performed in triplicates.

Our results indicated that the HPLC system is a solid method to study peptide acetylation. Using the non-radioactive HPLC method to detect the amount of acetylated oligopeptides, we determined hNaa30p enzyme kinetic constants for some *in vitro *oligopeptide substrates. Different concentrations of _1_MLGTG-RRR_24 _peptides (30–350 μM) were used with fixed concentration of acetyl CoA (300 μM) and 80 nM of purified MBP-hNaa30p. When using the absorption signal at 215 nm, representing acetylated oligopeptides, we calculated the K^_m oligopeptide_^ to be 283 μM with Vmax of 3.3 pmol * min^-1 ^* pmol hNaa30p^-1 ^(Table 2). In the same run, the production of CoA was recorded at 260 nm and used to calculate the corresponding kinetic constants. Here we observed that the Michaelis Menten plot based on CoA production generated a dose dependent curve from which a significantly lower Vmax was obtained (Figure 6) compared to when Vmax was calculated based on the production of acetylated oligopeptides (Figure 7). K_m oligopeptide _based on CoA production was determined to be 3.1 μM with a Vmax of 8.7 pmol * min^-1 ^* pmol hNaa30p^-1 ^(Table 2). It is important to note that approximately eight pmoles CoASH (Figure 6) were produced per pmole acetylated oligopeptide (Figure 7). Since theoretically one mole CoASH should be generated per mole acetylated oligopeptide, a discrepancy in CoASH production relative to acetylated oligopeptide was apparent. Km for acetyl CoA were with non linear regression determined to be approximately 14 μM with a Vmax of 2.1 pmol * min^-1 ^* pmol hNaa30p^-1 ^(Figure 8). V/K for selected substrates was calculated (Figure 9) and the S.D., indicated by error bars, were determined based on three independent experiments. The data demonstrate that substrate selectivity of the enzyme can be readily detected by the HPLC method.

**Table 2:** Comparison of K_m oligopeptide_ and Vmax based on the detection of CoA_(260 nm) _and the detection of acetylated oligopeptides_(215 nm)_.

CoA		Ac-_1_MLGTG-RRR_24_	
Km (μM)	Vmax (pmol product * min^-1 ^* pmol hNaa30p^-1^)	Km (μM)	Vmax (pmol product * min^-1 ^* pmol hNaa30p^-1^)

3.1	8.7	283.2	3.3

## Discussion and conclusion

The radioactivity-based filter assay [3] and the CoA-NADH coupled enzyme assay [5] are the most commonly applied methods to study acetyltransferase kinetics and mechanisms. Both these methods suffer from significant drawbacks such as biohazard and non-enzymatic deacetylation of acetyl CoA. To eliminate these problems and to allow us to analyse the acetylated oligopeptide products by mass spectrometry, we developed a method for studying peptide acetylation based on reverse phase HPLC. After acetylation, non-acetylated and acetylated oligopeptides are separated and quantified by integrating the respective elution peaks.

Since the first residues of the substrates seem to be most important for enzyme specificity [1], we designed peptides that deviated only within the 7 first N-terminal positions. The next 17 amino acids that are indicated by 'RRR' are identical in all peptides and resemble the sequence of Adrenocorticotropic hormone (ACTH) (Amino acid no. 8 to 24), but all Lys residues were replaced with Arg to minimize aberrant N-ε acetylation. The separation of peptides were carried out with 0.1% TFA in the HPLC buffers, making the peptide residues highly protonated. The positively charged Arg residues facilitate peptide solubility and separation by reverse phase HPLC. The N-terminal acetylation substitutes a positive charge by a hydrophobic group, causing the acetylated oligopeptides to be separated from the non acetylated form due to stronger interaction with the Nucleosil C18 HD matrix, resulting in increased elution time. Several acetylation assays with other N^α^-acetyltransferases acetylating oligopeptides containing more hydrophobic residues, showed that the acetylated form of these oligopeptides also could be efficiently separated with the reverse phase Nucleosile C18HD column. This indicates that commercially available oligopeptide substrates, composed of the endogenous amino acids, can be used as substrates with this detection method.

The unacetylated and acetylated oligopeptide _1_MLGTE-RRR_24 _was separated by more than 3 minutes. Even when using high amounts of oligopeptides, >30 nmols, leading to a widening of the peaks, an efficient separation of unacetylated and acetylated oligopeptides was achieved. If necessary, the separation of oligopeptides can be further enhanced by optimising the elution profile.

The sensitivity of the detection method was determined by injecting different amount of oligopeptides and acetyl CoA and quantifying the resulting absorption profiles. This showed a clear linear trend for both substrates, spanning from 0.5 to 5 nmol of acetyl CoA and from 1 to 10 nmol of oligopeptide. The coefficient of determination (R^2^) was above 0.97 for both substrates. Our experience with this acetylation assay is that even at very high amounts of oligopeptides, up to 30 nmol, the increase in absorbance at 215 nm is linear with coefficient of determination above 0.97 (data not shown).

The reproducibility of the method was analysed by calculating the standard deviation (S.D.) from three independent experiments. This was done for the sensitivity determination and the calculation of the kinetic constants. The result demonstrated that the reverse phase HPLC method is highly reproducible when analysing acetylation based on the detection of acetylated oligopeptides with the Abs 215 nm signal.

In conclusion, we have established a robust and highly reproducible method for studying oligopeptide acetylation. With new semi-automated reverse phase HPLC technology, we show that both substrates, acetyl CoA and unacetylated oligopeptides, and enzyme products, CoA and acetylated oligopeptides can be detected and quantified in the same experiment. This allows for increased control over substrate conversion and product generation forming a solid basis for data intepretation. Importantly, the assay is easy to perform and automation reduces sample handling.

## Competing interests

The authors declare that they have no competing interests.

## Authors' contributions

RHE and JRL designed the study. MZ and RHE proposed and optimized the method. RHE and KH conducted the experimental work. RHE and JRL participated in the data analysis and writing of the manuscript. All authors have read and approved the final manuscript.
